# Effects of Deep Brain Stimulation in the Subthalamic Nucleus on Neurocognitive Function in Patients With Parkinson's Disease Compared With Medical Therapy: A Meta-Analysis

**DOI:** 10.3389/fneur.2021.610840

**Published:** 2021-03-02

**Authors:** Jiazhen Wang, Ru Pan, Ying Cui, Zhigang Wang, Qinghua Li

**Affiliations:** ^1^Department of Neurology, Affiliated Hospital of Guilin Medical University, Guilin Medical University, Guilin, China; ^2^Pathology Department of Huzhou Central Hospital, Huzhou, China; ^3^Department of Neurology, Xiangya Hospital of Central South University, Changsha, China; ^4^Guangxi Clinical Research Center for Neurological Diseases, Affiliated Hospital of Guilin Medical University, Guilin Medical University, Guilin, China

**Keywords:** Parkinson's disease, deep brain stimulation, cognition, subthalamic nucleus, meta-analysis

## Abstract

**Background:** DBS has been shown to significantly affect motor symptoms in Parkinson's disease (PD). However, some studies have suggested that it may have adverse effects on patients' neurocognitive function. To clarify this operation's effect on neurocognitive function, we collected studies containing neurocognitive function evaluation for qualitative and quantitative analysis.

**Methods:** We searched relevant clinical studies through Pubmed and Embase databases and extracted and sorted out information such as sample size, post-operative scores, pre-operative and post-operative evaluation interval, PD course, and exclusion criteria, from articles meeting the standards. The magnitude and variance of the DBS group's combined effects and the drug therapy group in each neurocognitive domain were calculated and analyzed by the random-effects model.

**Results:** Compared with the drug treatment group, the verbal fluency of patients in the experimental group was significantly decreased at least moderately (ES = −0.553), in which the phonemic fluency declines greatly (ES = −0.842), learning and memory ability was slightly decreased (ES = −0.305), and other neurocognitive functions were not significantly decreased.

**Conclusion:** STN-DBS can affect verbal fluency and damage learning and memory. There was no significant correlation between the above effects and disease progression itself, and it was more likely to be associated with STN-DBS. It is suggested that post-operative patients should be trained and evaluated regularly for their verbal fluency and learning and memory ability. The safety of STN-DBS is acceptable for the majority of patients with motor symptoms.

## Introduction

Parkinson's disease (PD), also known as paralysis agitans (1), is a complex central neurodegenerative disorder characterized by motor and non-motor symptoms that affect an estimated 6.8 million people worldwide (2). The incidence of PD is rising faster than other neurodegenerative diseases (3). In the treatment of PD, early use of drugs can achieve a satisfactory effect (4). However, medication alone can only last for a few years; in the late stages of the disease, motor symptoms can be difficult to control with drugs (5). For this condition, Deep Brain Stimulation (DBS) could obtain good therapeutic effect (6, 7).

DBS is a neurosurgical procedure in which electrodes are specifically implanted into the brain area to improve motor symptoms, such as slowness and tremor, in PD patients (8–10). Currently, the most common target of DBS for PD patients is the bilateral subthalamic nucleus (STN) (11). STN-DBS surgery can significantly improve patients' motor symptoms (12) and reduce the need for medication (10). Although the benefits of motor symptoms in patients after STN-DBS have been widely recognized, this therapy's effect on neurocognitive outcomes remains controversial. Neurocognitive impairment is quite common in PD patients (13). Some patients with PD develop neurocognitive impairment before or at the time of diagnosis (13, 14), and their quality of life is affected by it (15). In a previous study, 49 PD patients were to receive bilateral STN-DBS, and the neurocognitive scale evaluated the neurocognitive function of the patients before and after the operation (16). After the evaluation, it was found that STN-DBS did not change PD patients' overall cognitive ability. However, some studies have reported different views. The meta-analysis by Parsons et al. evaluated the neurocognitive function of patients after STN-DBS operation by incorporating the data from 28 articles which met the eligibility criteria (17). The results showed that STN-DBS surgery had a certain impact on patients' verbal fluency. Weaver et al. found that neurocognitive functions, such as working memory and phoneme fluency, were slightly impaired after STN-DBS (18). At present, the neurocognitive effect of STN-DBS is still unclear. Further comprehensive and detailed analysis is needed to capture the full effect of STN-DBS better.

Most of the current studies did not design a control group, so the influence of disease progression itself cannot be ruled out. All the included studies included a drug control group, and neurocognitive function was evaluated by neurocognitive scale. This study aims to clarify the effect of STN-DBS on neurocognitive function and provide help and advice on clinical decision-making and post-operative rehabilitation based on its clinical importance. This study has been registered with PROSPERO, CRD:42020179012.

## Methods

### Search Strategy and Inclusion Criteria

The purpose of this study was to investigate the effects of subthalamic nucleus DBS on neurocognitive function in patients with PD. We conducted systematic retrieval in PubMed and Embase databases to find relevant clinical research papers. We used the following keyword search strategies: “Deep Brain Stimulation” its own words and MESH words, “PD” its own words and MESH words, “Cognitive OR Neuropsychological.” Two reviewers conducted the retrieval independently, by importing the retrieved literature into endnote and removing the duplicate literature. Based on the content of titles and abstracts, literature that might meet the inclusion criteria were acquired to identify eligibility. References of included literature were read to obtain relevant materials. The process was repeated until no relevant literature was found. Disagreements were anticipated to be settled by discussion or by a third evaluator. To be eligible for inclusion, a study needed: (1) Bilateral STN-DBS, (2) A drug control group, (3) Report of interval or digital data, (4) Use of at least one standardized neuropsychological scale, (5) Sufficient research results reported to calculate the effect size, and (6) Interventions that affect the results were not involved. Unpublished sources were not considered in this study.

### The Data Collection

After the implementation of the above criteria and discussion, two researchers independently extracted and collated the following information from the articles meeting the criteria: (1) sample size; (2) pre-operative and post-operative evaluation interval; (3) patient characteristics (such as Hoehn & Yahr, unified PD score scale); (4) The duration of PD; (5) education level; (6) the equivalent dose of levodopa of PD's drug; (7) surgical site; (8) sex; and (9) exclusion criteria (Table 1) (19–24). Neurocognitive tests were categorized into the following eight neuropsychological domains: cognitive screening, attention/concentration, executive functions, psychomotor speed, learning and memory, visuospatial skills, language, and verbal fluency (phonemic and semantic fluency). Since most neuropsychological scales involve multiple neurocognitive fields simultaneously, we divided each evaluation scale into subscales, which can correspond to a single neurocognitive field, and then assigned them to the main fields related to them (Table 2). This processing mode avoids the overweighting effect caused by the overlapping results of nerve scales across multiple domains. When the data arrangement was completed by the first author, the two investigators would negotiate to resolve data sorting and classification differences.

**Table 1:** Summary of studies included in the meta-analysis.

**References**	**Follow-up time**	**Exclusion criteria**	**Target Site**	**Unilateral/ Bilateral**	**Patients no. (STN/MMPD)**	**Age (STN/MMPD)**	**Gender STN:M/F MMPD:M/F**	**Education (y)**	**Disease duration M (SD) (STN/MMPD)**	**Mean L-dopa equivalents (STN/MMPD) (mg/day)**	**H&Y staging (STN/MMPD)**	**UPDRS scores III on (STN/MMPD)**	**UPDRS scores III off (STN/MMPD)**	**AAN class of evidence**
Tramontana et al. (19)	12	Secondary parkinsonism, dementia, Previous brain operation or injury. Active participation in another clinical trial for the treatment of PD. dyskinesias or motor fluctuations.	STN	Bilateral	15/15	60(6.8)/60 (7.0)	14/1 13/2	-	2.2 (1.4) 2.1 (1.1)	417.2 (306.6) 494.0 (208.7)	-	11.1 (6.9) 12.3 (6.4)	25.3 (9.0) 25.6 (5.8)	III
Zangaglia et al. (20)	36	Presence of dementia or psychiatric disease, no general surgical contraindications	STN	Bilateral	32/33	58.84 (7.70)/62.52 (6.82)	18/14 20/13	7.31(3.21) 7.58 (3.55)	11.84(5.07) 9.97 (4.86)	932.94 (409.86) 1043.51 (304.87)	3.2(0.67) 3.0 (0.7)	18.03 (8.34) 19.16 (8.0)	40.06 (15.53) 34.97 (12.15)	III
York et al. (21)	6	Presence of psychiatric complications that interfere with compliance, MMSE Score <24, H&Y “on” score stage 5, Medical contraindications,	STN	Bilateral	23/27	59.5 (11.8)/66.7 (8.7)	13/10 20/7	14.4 (2.6) 16.3 (1.3)	11.6 (9.4) 4.7 (4.4)	1009.8 (445.2) 358.9 (287.0)	2.27 (0.42) 2.13 (0.58)	21.1 (11.5)	49.3 (11.3)	III
Girronell et al. (22)	6	Dementia, major depression, marked cerebral atrophy	STN	Bilateral	8/8	56.6 (4.8)/55.8 (7.2)	-	9.4 (5.6) 8.3 (2.2)	12.5 (4.8) 11.7 (4.7)	1020.0 (490.2) 995.3 (340.3)	4.3 (0.6) 4.2 (0.7)	-	59.9 (15.5) 55.2 (8.7)	III
Rinehardt et al. (23)	5	Prior history of psychiatric disease, Prior diagnosis of another neurological disease or dementia	STN	Bilateral	20/20	66.7 (9.4)/69.3 (6.5)	10/10 18/2	13.4 (2.3) 12.1 (2.8)	9.4 (5.1) 7.5 (5.9)	-	3.2 (0.5) 2.6 (0.5)	-	-	III
Williams et al. (24)	24	MMSE Scores <23 and presence of psychiatric complications that could interfere with compliance	STN	Bilateral	19/18	62.1 (10.3)/66.6 (9.0)	10/9 15/3	13.6 (1.7) 16.6 (1.20)	10.1 (6.2) 7.50 (4.22)	1017.6 (411.2) 468.4 (293.0)	-	-	-	III

*PD, Parkinson's disease; DBS, deep brain stimulation; MMSE, Mini-Mental Status Examination; UPDRS, Unified Parkinson's Disease Rating Scale; H&Y, Hoehn and Yahr; MMPD, Medically Managed Parkinson's disease patients; STN, Subthalamic Nucleus*.

**Table 2:** Tests included in each neurocognitive domain.

**Domains**	**Neuropsychological test**	***K***	***n***	***Q***
Cognitive screen	Dementia rating scale	5	87	3.89^*^
	Mini mental status exam		152	
	Wisconsin card sorting test-Categories		46	
Attention and concentration	Attentive matrices	5	16	4.65^*^
	Corsi's block tapping test		65	
	Digit span		192	
	Verbal span		65	
Executive functions	Trail making test B	5	103	10.69
	Wisconsin/Modified card sorting tasks		198	
	Stroop Color/Word		67	
	Stroop interference		66	
Psychomotor speed	Stroop color naming	4	46	2.12^*^
	Stroop word reading		133	
	Symbol digit modalities test		87	
	Trail making test A		103	
	WAIS digit symbol coding		30	
Learning and memory	WMS-III word list learning	4	30	0.39^*^
	WMS-III memory for faces		30	
	Rey auditory verbal learning test		103	
	Brief visuospatial memory test Benton		103	
Visuospatial skills	Judgment of Line Orientation	4	30	6.3
	RBANS: Line orientation		40	
	RBANS: Figure copy		40	
	Clock command		87	
Language	Boston naming test	3	117	3.86^*^
Verbal fluency	Phonemic fluency	5	198	5.21^*^
	Semantic fluency	5	173	

*K, number of studies evaluating the cognitive domain; Q, Cochran's Q statistic; WAIS, Wechsler adult intelligence scale; PASAT, paced auditory serial addition task; WMS, Wechsler memory scale; RBANS, Repeatable Battery for the Assessment of Neuropsychological Status*.

* *p > 0·10*.

### The Data Analysis

The random-effects model was used in this study because there are many differences between the study samples. The total effect obtained by this method represents the population mean of the real effect. In each study, to evaluate the effect of STN-DBS on the neurocognitive function of patients, various neurofunctional scales were used to test the patients. There was no uniformity between the results of different scales. To compare the results between different scales and different studies, we chose the standardized mean difference (also known as Cohen's d) as the indicator of effect size. The standardized mean difference is an index that can be compared between different studies. Its calculation formula is $d=\frac{Y_{1}-Y_{2}}{S_{within}}$ (*Y*_1_ and *Y*_2_ are the mean values of the two groups, *S*_*within*_ is the standard deviation within the group). A negative d value indicates a decrease of a certain index after DBS surgery, whereas a positive d value indicates an improvement of a certain domain after DBS surgery. It seems that we can directly meta-analyze each subscale data in different studies in different neurocognitive fields as an independent result. However, this will cause a problem; when calculating the comprehensive effect of all studies, this method will assign more weight to research with more results, which will lead to an incorrect estimation of the precision of the comprehensive effect. So we need to calculate the mean effect size of each study's multiple outcomes in each neurocognitive domain, and then apply that mean size as a unit of analysis, rather than treating each outcome as a separate unit of analysis (25). The formula for calculating the mean value of the effect size is $D=\frac{\sum_{i=1}^{n}d_{i}}{n}$, where *d*_*i*_ represents the effect value of the *i*th subscale in a certain neurocognitive field of the paper. The next step is to calculate the variance of each d value, the calculation formula is $V_{d}=\frac{\left(n_{1}+n_{2}\right)}{\left(n_{1}n_{2}\right)}+\frac{d^{2}}{2\left(n_{1}+n_{2}\right)}$, *n*_1_ and *n*_2_, respectively represent control group's sample size and the experimental group in the study. We also need to calculate the variance of the mean effect size for each article in each field. The formula for calculating the variance of mean effect size is $V_{d}={\left(\frac{1}{n}\right)}^{2}\left[\overset{n}{\underset{i=1}{\sum }}V_{i}+\underset{i\neq j}{\sum }\left(r_{ij}\sqrt{V_{i}}\sqrt{V_{j}}\right)\right]$, where r is the correlation coefficient, which describes the degree of covariation between *V*_*i*_ and *V*_*j*_, ranging from 0 to 1. In this study, the median value is 0.5 and *V*_*i*_ represents the variance of the effect magnitude value of the *i*th sub-scale in a certain field. The average synthetic unbiased effect M was obtained by calculating the mean effect size and its variance under the random effect model. According to Cohen's suggestions, an effect size of 0.20 was regarded as a small effect, 0.50 as a moderate effect, and 0.80 as a large effect.

To test whether there are differences among the study samples, we conducted a homogeneity test on the effect sizes of each neurocognitive domain, enabling the authors to determine the homogeneity of the included studies. Next, the mean weighted effect size, standard error, and 95% confidence interval for each neurocognitive region were calculated. Stata and Maple software were used for the above calculation and analysis.

## Results

### Characteristics of Eligible Studies

**Figure 1:**
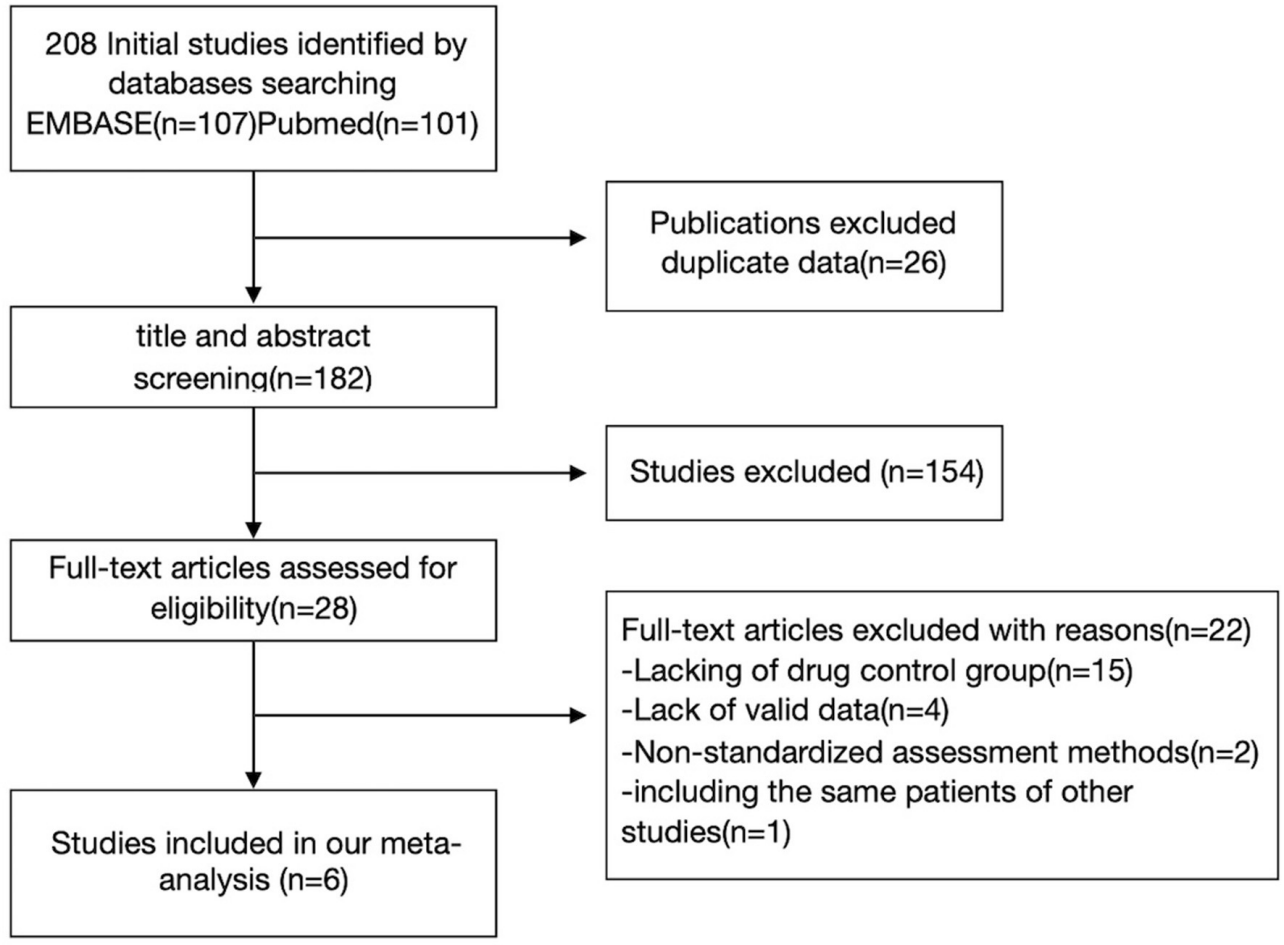
Flow diagram illustrating the systematic search strategy and review process that was used to identify the articles included in the review.

We retrieved 208 articles related to the above keywords, and 28 articles related to this study were obtained by reading the titles and abstracts. The full text of these 28 articles was read, and after the exclusion of 22 articles, the remaining six articles met the inclusion criteria of this meta-analysis. Twenty two articles were excluded from the analysis for the following reasons: lack of a control group (*n* = 15); Lack of standardized assessment methods (*n* = 2); Insufficient information was provided for effect size calculation, and the author could not be contacted for data (*n* = 4); and Duplicate data (*N* = 1). Figure 1 shows a flowchart of the filter method. In each neurocognition field, the maximum combined sample size used to calculate the aggregate effect size was 434 cases. The AAN (American Academy of Neurology) was used to evaluate the study quality of the included study. The literature's randomization method, allocation and hiding scheme, blindness, completeness of result data, selective reporting of study results, and other biased sources were evaluated (Table 1).

### Quantitative Synthesis

The heterogeneous sizes *Q* and *P*-values of the various neurocognitive domains are listed in Table 2 to determine whether the studies in these domains are homogeneous enough to function as appropriate independent entities. In the field of spatial visualization ability, heterogeneity among merged literature was Cochran's *Q* = 6.30, *P* = 0.098, I^2^ = 52.4%. This suggests that some of the effector results in this area are due to differences between the combined studies. To clarify the possible sources of heterogeneity, we conducted subgroup analysis on factors, such as the year of publication, type of study, duration of follow-up, and course of PD, of the above studies, but no obvious source of heterogeneity was observed. Similarly, there is considerable heterogeneity in the executive function domain results, Cochran's Q = 10.69, *P* = 0.030, I^2^ = 62.6%, No possible source of heterogeneity was observed in subgroup analysis. After careful discussion, we decided to use the random-effects model to attenuate heterogeneity and hypothesize that inclusion studies' different exclusion criteria might be the cause of heterogeneity. The Cochran *Q*-value in the other fields was not significant (*p* > 0.10), indicating that the studies combined in the other fields had good homogeneity, and the results were relatively robust.

#### Combined Effect Sizes Were Calculated for Each of the Eight Neurocognitive Domains

Since the verbal fluency test requires an individual to produce as many words that begin with a particular letter (Phonemic fluency) or belong to a certain category (Semantic fluency) (26) as possible in a limited time, the effects in the verbal fluency domain are based on the merging of phonemic and semantic fluency subdomains. Evaluations on the comprehensive effect after the combination, and giving priority to the areas with significant clinical effects. Patients who underwent STN-DBS experienced a slight decrease in learning and memory compared to the drug treatment group (ES = −0.305, 95%CI:−0.595~-0.014, *P* = 0.040). The results also showed a moderate decline in verbal fluency (ES = −0.553, 95%CI:−0.798~-0.309, *P* = 0.000), a large degree of decline in phonemic fluency (ES = −0.842, 95%CI: −1.135~-0.550, *P* = 0.000), and a moderate decline in semantic fluency (ES = −0.405, 95%CI:−0.757~-0.053, *P* = 0.024). There were no observable changes in other areas of neurocognition (Table 3).

**Table 3:** Random effect sizes for the neuropsychological domains.

**Domain**	**Average random effect size**	**SE**	**95% CI**
Cognitive screen	−0.106	0.167	(−0.434, 0.221)
Attention and concentration	−0.251	0.144	(−0.534, 0.032)
Executive functions	−0.025	0.209	(−0.434, 0.384)
Psychomotor speed	−0.197	0.144	(−0.480, 0.086)
Learning and memory	−0.305^*^	0.148	(−0.595, −0.014)
Visuospatial skills	−0.124	0.227	(−0.568, 0.321)
Language	−0.379	0.265	(−0.898, 0.139)
Verbal fluency	−0.553^*^	0.125	(−0.798, −0.309)
Phonemic fluency	−0.842^*^	0.149	(−1.135, −0.550)
Semantic fluency	−0.405^*^	0.179	(−0.757, −0.053)

* *Function was decreased after deep brain stimulation of the subthalamic nucleus for Parkinson's disease*.

## Discussion

This meta-analysis conducted data statistics and analysis on six included articles. Among them, five were non-randomized controlled studies, and one was a randomized controlled study. In these studies, PD patients were divided into a drug therapy group and an STN-DBS group. During the follow-up, the general scale was used to evaluate and track the patients' neurocognitive function. The results were different between them. Most studies indicate that the verbal fluency of patients after STN-DBS is decreased, especially semantic fluency. Impairments in learning and memory, executive function, and information processing have been reported in different studies. To further clarify the effect of DBS on the neurocognitive function of patients, we combined the above study data for analysis. The analysis results show that, compared with the drug treatment group, patients' verbal fluency after STN-DBS showed a moderate or significant decrease (ES = −0.553). There was a slight decrease in learning and memory ability (ES = −0.305), but no significant decrease in other neurocognitive functions was observed. Among them, verbal fluency can be subdivided into semantic fluency and phonemic fluency, which declines to a large extent (ES = −0.842) and a moderate extent (ES = −0.405), respectively.

Due to the limitations of the meta-analysis, the results might be affected in many ways. Therefore, we must interpret the results of this meta-analysis carefully. The quality of the included studies limits the results' reliability, and we address this by setting certain inclusion criteria. As with any research review in a particular field, studies with insignificant results will likely encounter more resistance to publication. The practice of publishing only studies with significant results may bias the study subjects' results, especially in the case of meta-analysis, which is likely to lead to publication bias. Since not every study contains data from eight neurocognition areas, the results of a single neurocognition field come from the summary of some research evaluations.

Caution is also needed in interpreting the clinical significance of effector quantities. In particular, the magnitude of the effect size itself is not absolute, and a small difference in size does not mean that these events differ in clinical practice. Besides, the magnitude of effect size is not consistent with its clinical significance. An event with an effect of 0.15 and *P*-value < 0.05 will be described as a small but significant effect statistically. However, the clinical significance depends on the event's importance, so the statistical significance is different from the clinical significance, and the effect size cannot be mechanically equal to the clinical significance.

### Verbal Fluency Drop

Verbal fluency, which is composed of semantic and phonemic fluency, was often used to evaluate the level of neurocognitive function in patients (27). A semantic fluency test requires individuals to generate as many words as possible belonging to a certain category (e.g., animals) in a limited time; for a phonemic fluency test, the words produced must begin with a specific letter (e.g., S) (28). These measures are considered to reflect the ability of verbal retrieval and recall, as well as self-monitoring aspects of cognition (29). From the results of this study, we can see that, compared with the drug group, the decrease of verbal fluency in the operation group is obvious. The decline of phonemic fluency is the most obvious. This suggests that there may be no significant correlation between the decline of this function and the progression of the disease. STN-DBS may be the main cause of this phenomenon (30, 31). Studies have reported that impaired frontal lobe function is associated with decreased language fluency (32). Marshall and his team found a significant decrease in verbal fluency after STN-DBS; this change is related to the decrease of information processing speed, and it may be caused by the frontal lobe function changes caused by STN-DBS (33). Along with this point of view, the decline of verbal fluency might be caused by physical damage to the electrodes, electrical discharge stimulation of the electrodes, or a combination of the two, all of which need to be further elucidated (34–36).

From the perspective of neuroanatomy, a variety of white matter neural pathways were located in the electrode implantation track, and the electrode might damage their anatomical structures. The implantation effect of the surgery might play an important role (37, 38). The study by York et al. indicated that patients' decreased verbal fluency after STN-DBS was related to the surgical trajectory and electrode placement (39). Costentin et al. analyzed 48 patients who underwent bilateral STN-DBS. They converted the electrode insertion locus into a 3D image and compared it with the white matter bundle map, such as the frontal striatum tract and anterior thalamic tract. However, the relationship between impaired white matter neural pathways and decreased semantic and phonemic fluency has not been proven (40). Due to the lack of more necessary data, they couldn't make it clear whether the decline in verbal fluency might not be related to a single specific pathway but instead to simultaneous damage to several major pathways in the brain. Although there is currently no clear evidence to support the link between decreased verbal fluency and physical damage from electrode implantation, this is still a promising direction for exploration. As more clinical trials are carried out and more data are analyzed, the relationship between physical electrode damage and decreased verbal fluency will become clearer.

The electrical stimulation parameters after electrode implantation may affect the verbal fluency of patients (41–47). For example, Fagundes' team observed that the discharge frequency of electrodes affected the verbal fluency of patients. The researchers scored the verbal fluency under low frequency (60 Hz) and high frequency (130 Hz) stimulation, respectively. The results showed that the damage of high-frequency stimulation to verbal fluency was more obvious than that of low-frequency stimulation. The decrease of phonemic fluency in sub-domains was more obvious (41). Lars Wojtecki et al. reported similar results when they adjusted the frequency of electrical stimulation at non-stimulation, 10 Hz, and over 130 Hz in 12 patients after STN-DBS surgery; verbal fluency was assessed 5 min after each stimulation. The results showed that, compared with no stimulation, patients' verbal fluency showed a trend of improvement at 10 Hz, but there was no statistical significance. The verbal fluency was worsened by over 130 Hz's stimulation, and the results were statistically significant (44). Many studies have shown that, although high-frequency stimulation effectively improves motor symptoms, it seems to have the side effect of damaging verbal fluency. There are few studies on the influence of stimulus amplitude and pulse on verbal fluency. A prospective clinical trial by Schoenberg et al. found that larger electrical stimulation amplitudes and pulse widths improved semantic fluency in patients (46). However, the sample size of this study was small, and stimulus parameters were not adjusted in a planned way during each evaluation, which may affect the reliability of the conclusion. Although there is little research in this area, the current results may have potential theoretical and clinical applications. To balance the motor symptoms and neurocognitive function of patients, we can try to make individualized settings of electrical stimulation parameters, especially the stimulation frequency, on the premise of ensuring the obvious remission of motor function. The association between electrical stimulation parameters and verbal fluency has not yet been satisfactorily explained and needs to be verified in more basic and clinical trials (47).

### Other Neurocognitive Implications

The study also found that STN-DBS had a mildly negative impact on patients' learning and memory ability than the drug treatment group. Mayer and his team have shown that STN-DBS leads to a deterioration in trend levels of learning and memory performance, perhaps caused by DBS affecting the basal ganglia-thalamocortical circuits (48). This is consistent with the results of this study. However, some studies have not observed the above conclusions. Merkl and his team reported that STN-DBS had no significant detrimental effect on this area of neurocognition (49). The effect-value index of learning and memory ability decreased during electrical stimulation, but the statistical results were not significant (50). The above studies' results were not included in this analysis because they were not data from a standardized neurocognitive scale. The different conclusions may be caused by the sampling error and systematic error in the research process due to the research methods and evaluation methods. Other variables, such as dosage, disease severity, and major symptoms, can also complicate the results. We cautiously put forward a point of view that STN-DBS may cause mild damage to the ability of learning and memory and has no significant impact on the post-operative short-term quality of life. However, a pre-operative evaluation of relative contraindications may be required in patients with severe symptoms who plan to undergo STN-DBS.

### The Limitations and Future Directions

This study has some limitations, and these limitations should be recognized and solved in future research. The number of studies included in this study is not sufficient, and the number of studies further decreased after being subdivided into various neurocognitive fields. Therefore, meta regression is not conducted in this study because too few studies would lead to great uncertainty in the results. With the increase of the number of follow-up studies meeting the criteria, the effects of other variables, such as duration of PD, follow-up time, and equivalent dose of levodopa on various neurocognitive domains, can be understood through meta regression. Also, the proportion of randomized controlled trials in the included literature is relatively low, which may influence the research results. In future studies, we need to include more high-quality randomized controlled studies to improve the conclusions' reliability. In addition, it is worth noting that the scales used to assess patients' neurological function are reused, which may lead to a learning effect, and some small changes may be masked. It is hoped that scales can evaluate patients with similar efficacy but different content in the future.

In the included studies, some did not record the correlation coefficient r between different outcome variables. Therefore, we cannot accurately calculate the intergroup variance of each outcome. The accuracy of the binding effect in a single field will be slightly affected, and its positive and negative properties and statistical significance will not be affected. In future clinical trials, it is better for the experimenters to provide the correlation coefficient between the scales and reduce the disturbance of the small but important clinical significance fields by improving combined results' accuracy.

## Conclusion

STN-DBS has some impact on the neurocognitive function of patients; it is mainly reflected in the moderate decrease of verbal fluency, in which the phonemic fluency declines greatly. The learning and memory function of the patients had a potential trend of weakening, but it was not statistically significant. These decreases are associated with STN-DBS, but the mechanism has not been clearly elucidated. In most cases, these effects can be accepted as a result of the substantial motor improvement. In the future, we need to further clarify DBS's mechanism and make a more individualized formulation of patients' stimulus parameters. This would mean that DBS can be used to maximum effect, and not only in improving motor function, which is something greatly anticipated by researchers.

## Data Availability Statement

The raw data supporting the conclusions of this article will be made available by the authors, without undue reservation.

## Author Contributions

JW: conceptualization, methodology, software, formal analysis, data curation, and wrote-original draft. RP: conceptualization, data curation, wrote-original draft, and wrote-review and editing. YC: wrote and revised the manuscript. ZW: wrote-original draft and wrote-review and editing. QL: wrote-review and editing, visualization, supervision, and funding acquisition. All authors contributed to the article and approved the submitted version.

## Conflict of Interest

The authors declare that the research was conducted in the absence of any commercial or financial relationships that could be construed as a potential conflict of interest.
